# Cooperative growth of *Geobacter sulfurreducens* and *Clostridium pasteurianum* with subsequent metabolic shift in glycerol fermentation

**DOI:** 10.1038/srep44334

**Published:** 2017-03-13

**Authors:** Roman Moscoviz, Florence de Fouchécour, Gaëlle Santa-Catalina, Nicolas Bernet, Eric Trably

**Affiliations:** 1 LBE, INRA, 102 Avenue des étangs, 11100 Narbonne, France

## Abstract

Interspecies electron transfer is a common way to couple metabolic energy balances between different species in mixed culture consortia. Direct interspecies electron transfer (DIET) mechanism has been recently characterised with *Geobacter* species which couple the electron balance with other species through physical contacts. Using this mechanism could be an efficient and cost-effective way to directly control redox balances in co-culture fermentation. The present study deals with a co-culture of *Geobacter sulfurreducens* and *Clostridium pasteurianum* during glycerol fermentation. As a result, it was shown that *Geobacter sulfurreducens* was able to grow using *Clostridium pasteurianum* as sole electron acceptor. *C. pasteurianum* metabolic pattern was significantly altered towards improved 1,3-propanediol and butyrate production (+37% and +38% resp.) at the expense of butanol and ethanol production (−16% and −20% resp.). This metabolic shift was clearly induced by a small electron uptake that represented less than 0.6% of the electrons consumed by *C. pasteurianum*. A non-linear relationship was found between *G. sulfurreducens* growth (*i.e* the electrons transferred between the two species) and the changes in *C. pasteurianum* metabolite distribution. This study opens up new possibilities for controlling and increasing specificity in mixed culture fermentation.

To sustain their growth and maintenance, microorganisms perform oxidative and reductive reactions inside their cells. These redox reactions consist in electron flows coming from an electron donor that are stepwise transferred to a terminal electron acceptor (*e.g.* O_2_ in aerobic respiration) with an overall release of free energy. However, one single species is not always able to perform the entire cascade of reactions. In this case, some microorganisms couple their electron flows with other species via interspecies electron transfer (IET) to carry out reactions that would otherwise be thermodynamically unfavourable^1^,^2^. A well described example is the IET existing between archaea and bacteria during methanogenesis through the diffusion of H_2_ or formate^2^,^3^. More recently, direct interspecies electron transfer (DIET) that does not proceed through the diffusion of electron carriers has been discovered. During DIET, electrons are transferred via biological electrical connections between electron-donor (exoelectrogens) and electron-acceptor (electrotrophs) microorganisms^2^,^4^,^5^. Contacts between the two partners can be ensured by the establishment of a biofilm on a conductive material^1^,^5^ (*e.g.* iron oxides or carbon materials) or by connecting species with pili with metallic-like conductive properties^1^,^2^,^4^. These pili, named nanowires, can be produced by iron-reducing bacteria such as *Geobacter metallireducens*^6^ or *Geobacter sulfurreducens*^7^ that are even able to connect bacteria up to a centimetre scale^8^. Instead of using Fe^3+^, these species were reported to be able to transfer their electrons to other species, such as denitrifying bacteria^9^,^10^ or methanogens^11^.

Interestingly, one of the two partners of a DIET can be replaced by an electrode that act as an artificial electron donor^12^,^13^ (cathode) or acceptor^14^ (anode). This is the basis of bio-electrochemical systems (BESs), processes that have been designed to take advantage of electro-active bacteria to produce electricity, chemicals or other services^15^. For instance, exoelectrogens (*e.g. Geobacter* species) can generate electrical power in microbial fuel cells while oxidizing organic matter from waste^14^. Methanogens or denitrifying bacteria (electrotrophs) are also able to consume electrons from a cathode in microbial electrolysis cells to convert CO_2_ into methane^16^, or reduce nitrates, respectively^17^,^18^. The intensive research that has been conducted on BESs has revealed that besides the well-known *Geobacter* and *Shewanella* species, many other microorganisms are able to interact directly with electrodes^12^,^13^,^19^. In particular, metabolic patterns of fermentative bacteria could be affected by small input of electrons through a cathode during electro-fermentation experiments^20^,^21^. As an illustration, *Clostridium pasteurianum* was reported to be able to consume cathodic electrons during fermentation, and produced more butanol and 1,3-propanediol (PDO) from glucose and glycerol respectively than during classic fermentation^22^. As this fermenter was able to uptake extracellular electrons from a cathode, it is not excluded that electrons provided by DIET with an exoelectrogen organism could also be consumed and lead to a similar metabolic shift. To date, studies on electron flux between exoelectrogens such as *Geobacter* species and fermenters have always focused on the degradation of organic matter by the fermenters into simple carboxylic acids that could be readily converted by the exoelectrogens into electricity in microbial fuel cells, *i.e.* electron transfer from the fermenting to the electrogenic organism^23^,^24^.

The aim of this study is to provide a proof-of-concept experiment showing that electron coupling between fermenters and exoelectrogens is also possible the other way around: in this case, the fermentative species would be the electron acceptor while the exoelectrogens would provide electrons. This could serve to trigger a metabolic shift towards the production of more reduced products such as PDO. This experiment was conducted for glycerol fermentation using a defined co-culture of *G. sulfurreducens* and *C. pasteurianum* as model partners for DIET.

## Results

### Growth of *G. sulfurreducens* and *C. pasteurianum* in co-cultures

**Figure 1:**
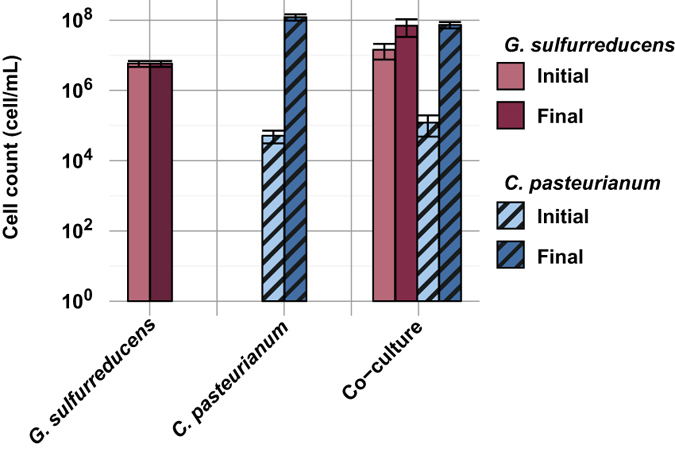
Growth of *G. sulfurreducens* and *C. pasteurianum* in the different experimental conditions. Cell counts are based on qPCR results and corrected by the respective number of copies of 16S rRNA for each strain. Error bars represent the standard deviation of the replicates.

To study the possible interactions that could exist between *G. sulfurreducens* and *C. pasteurianum*, the two strains were inoculated in a medium containing glycerol (fermentation substrate for *C. pasteurianum*) and acetate (electron donor for *G. sulfurreducens*). The growth of both *G. sulfurreducens* and *C. pasteurianum* were monitored using qPCR, as shown in Fig. 1. To evaluate the growth of a population, the number of generation (N_g_) can be used that corresponds to the log_2_ ratio of the final population count over the initial population count (see Equation 2). During pure culture of *G. sulfurreducens*, no growth occurred as initial and final cell counts were strictly identical (3.3 ± 1.9 10^6^ and 3.3 ± 1.2 10^6^ cells.mL^−1^ respectively, n = 8 replicates), resulting in N_g_ < 0.5 (twice the standard error of qPCR measurements). This confirmed that no electron acceptor was available in the fresh medium to sustain the growth of *G. sulfurreducens*. Pure cultures of *C. pasteurianum* were inoculated at 5.2 ± 2.0 10^4^ cells.mL^−1^ (n = 4). Growth was observed and stopped at 1.2 ± 0.2 10^8^ cells.mL^−1^ after total substrate depletion (N_g_ = 11.2 ± 0.35, n = 4).

Considering the co-culture experiments, *G. sulfurreducens* grew in only two of the four replicates. During these two replicates, a slightly lower growth of *C. pasteurianum* was observed when compared to the pure culture control (N_g_ = 9.3 ± 0.8, n = 2), with an inoculation at 1.2 ± 0.7 10^5^ cells.mL^−1^ and a final concentration of 7.4 ± 1.5 10^7^ cells.mL^−1^ (n = 2). Interestingly, a significant growth of *G. sulfurreducens* was also observed, with N_g_ = 2.2 ± 0.1 (n = 2). This means that *G. sulfurreducens* was able to use an electron acceptor that was not present in the fresh medium. The relatively low growth of *G. sulfurreducens* could be explained by its high doubling time (T_d_) as previously reported in electron acceptor limiting conditions (T_d_ = 6.93 h)^9^,^25^, especially when compared to *C. pasteurianum* doubling time in glycerol fermentation (T_d_ = 1.87 h)^26^. If *C. pasteurianum* accepts electrons from *G. sulfurreducens*, it can be hypothesized that G*. sulfurreducens* could only grow during *C. pasteurianum* growth phase. In this context, the ratio of generation numbers N_gGsul_/N_gCpast_ of G*. sulfurreducens* and *C. pasteurianum* respectively in co-culture should not exceed the ratio of the corresponding doubling times. During this experiment, the N_g_ ratio was 0.24 ± 0.04 and was comparable to the T_d_ ratio of 0.27 calculated from values reported in the literature, supporting the synchronous growth of G*. sulfurreducens* and *C. pasteurianum.*

### Metabolic patterns shifted during co-cultures

**Figure 2:**
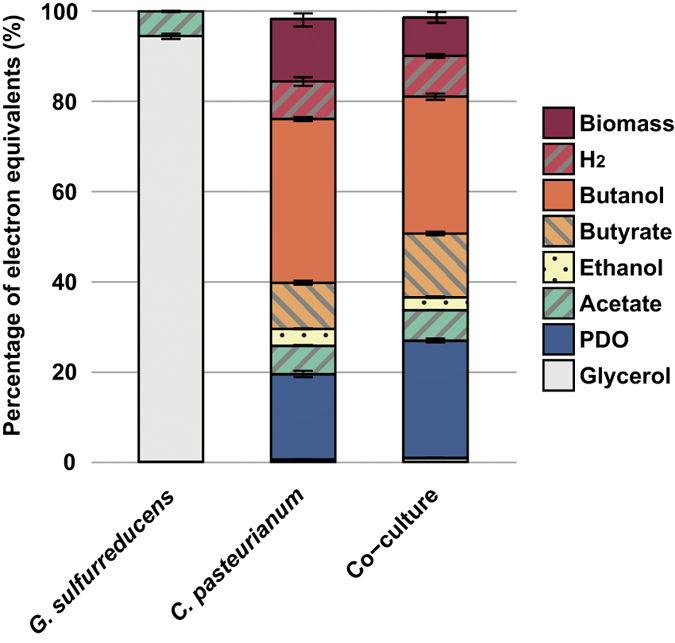
Electron mass balances calculated from the metabolites measured at the end of co-culture experiments. Results are normalized by the sum of electron content from initial glycerol and acetate. Biomass was calculated from qPCR cell count results. Error bars represent the standard deviation of the replicates.

After 72 h of fermentation, metabolites in the liquid phase were measured to establish mass balances. Electron mass balances closed between 98.3 and 100.0%, meaning that all major metabolic products were quantified (see Fig. 2). When *G. sulfurreducens* was cultivated alone (n = 8), no substrate was consumed. In contrast, all the glycerol was depleted after 72 h with the pure cultures of *C. pasteurianum* (n = 4). The main metabolite was butanol with a yield of 225 ± 5 mmol.mol_glycerol_^−1^. Other major metabolic products were 1,3-propanediol (PDO, 176 ± 13 mmol.mol_glycerol_^−1^), ethanol (54 ± 2 mmol.mol_glycerol_^−1^) and butyrate (76 ± 7 mmol.mol_glycerol_^−1^). Only the two replicates of *G. sulfurreducens* and *C. pasteurianum* co-cultures where *G. sulfurreducens* growth was observed had different metabolic patterns when compared to the pure cultures of *C. pasteurianum*. In these two replicates, PDO and butyrate yields significantly increased to 241 ± 8 mmol.mol_glycerol_^−1^ (p = 0.016) and 105 ± 6 mmol.mol_glycerol_^−1^ (p = 0.018) respectively, with regard to *C. pasteurianum* alone. On the opposite, butanol and ethanol yields significantly decreased to 188 ± 9 mmol.mol_glycerol_^−1^ (p = 0.016) and 43 ± 4 mmol.mol_glycerol_^−1^ (p = 0.023), respectively. In addition, samples were taken from the co-cultures after 240 h, to observe additional metabolite production or consumption following glycerol depletion. No significant changes were observed in comparison with the samples at 72 h. As electron donors (*i.e.* acetate) were still present in the medium, this observation confirms that *G. sulfurreducens* was not able to directly use the end-products issued from *C. pasteurianum* fermentation as electron acceptors.

### Metabolic shift of *C. pasteurianum*

**Figure 3:**
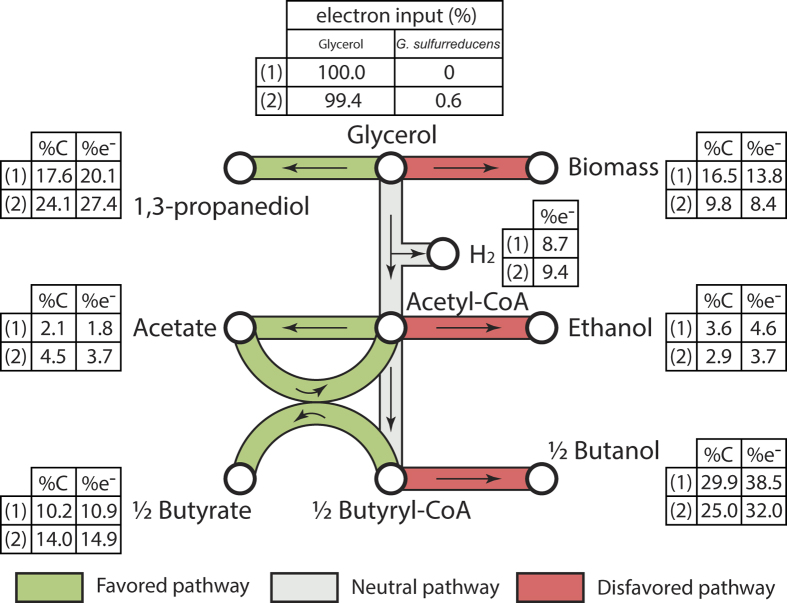
Average carbon and electron distributions of the products from glycerol fermentation during (1) pure cultures of *C. pasteurianum* and (2) co-cultures of *G.sulfurreducens* and *C. pasteurianum*. Pathways are considered to be favoured or disfavoured if more than 10% increase of decrease resp. was observed between (1) and (2).

Assuming that *G. sulfurreducens* transferred its electrons to *C. pasteurianum*, electron and carbon mass balances could be estimated for *C. pasteurianum* only (see Fig. 3). Calculations were made under the following hypothesises: (i) acetate is the only carbon source and electron donor for *G. sulfurreducens* growth^27^, (ii) this species uses only 10% of the electron equivalents it consumed for its own growth^25^ and (iii) all the remaining electrons are transferred to *C. pasteurianum*. In this context, the acetate consumed by *G. sulfurreducens* for its own growth could be assessed from qPCR data and was estimated around 1.1 ± 0.4 mM (n = 2). According to the different hypothesises, this acetate was used by *G. sulfurreducens* according to the following equation (1):


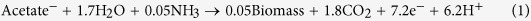


The electrons released from acetate consumption represented 0.6% of the total electron equivalents consumed by *C. pasteurianum*. If this small amount of electrons was directly dissipated by *C. pasteurianum* by converting glycerol into PDO (2 moles electrons consumed per mole PDO produced), it would increase the PDO production from 17.6%_total carbon_ (pure culture control) to only 21.5%_total carbon_. However, 24.1 ± 0.8%_total carbon_ (n = 2) was recovered as PDO in the co-culture. It was therefore concluded that a direct dissipation of the electrons provided by *G. sulfurreducens* could not be the unique reason of the changes in metabolic patterns. In fact, carbon and electrons were also diverted from biomass synthesis and solventogenesis pathways (*i.e.* production of ethanol and butanol) to the production of carboxylic acids and PDO (see Fig. 3). All these observations are consistent with previous electro-fermentation results reported by Choi *et al*.^22^. Providing a small quantity of electrons from a cathode to the same strain of *C. pasteurianum* (2% of the total electron input) resulted in a decrease of biomass and butanol production and an increase of PDO and butyrate production. Concerning biomass synthesis, this result is surprising since the increase and decrease in carboxylic acids and alcohols, respectively, should have theoretically led to an increase of the ATP production by 8.6%. How this extra ATP was dissipated remains unknown.

### Effect of *G. sulfurreducens* growth on *C. pasteurianum* metabolites yields

**Figure 4:**
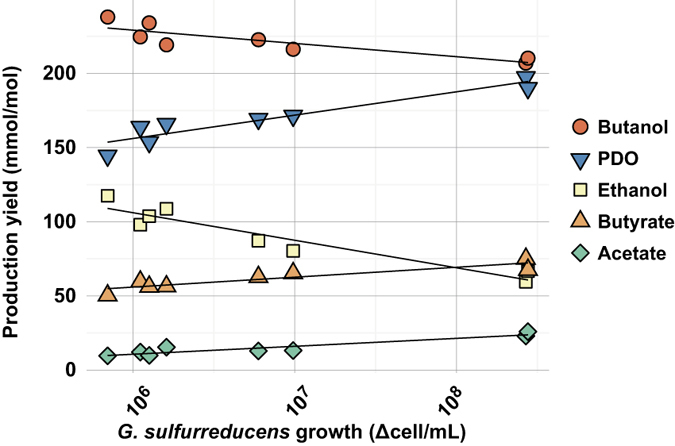
Production yields as a function of *G. sulfurreducens* growth during co-culture experiments with *C. pasteurianum*. Only the results of experiments with a significant *G. sulfurreducens* growth are displayed (N_g_ > 0.5 log_2_(cells).mL^−1^).

In order to study the dependence of *C. pasteurianum* metabolic shift on biomass production of *G. sulfurreducens*, three quadruplicate experiments were carried out with distinct *G. sulfurreducens* initial concentrations of 7.4 ± 2.8 10^5^ (n = 4), 8.9 ± 4.6 10^6^ (n = 4) and 1.1 ± 0.4 10^8^ (n = 4) cells.mL^−1^ respectively. In addition, four pure cultures of *C. pasteurianum* were performed as controls. All metabolite yields together with the growth yields of *C. pasteurianum* and *G. sulfurreducens* are provided in Table 1. Out of 12 co-cultures, only 8 experiments exhibited significant *G. sulfurreducens* growth (N_g_ > 0.5 log_2_(cells).mL^−1^). The average numbers of generation N_gGsul_ of *G. sulfurreducens* (N_gGsul_ = 1.4 ± 0.4, n = 8) was lower than in the previous experiment (N_gGsul_ = 2.2 0.1). The N_gCpast_ of *C. pasteurianum* was also lower (N_gCpast_ = 6.6 ± 0.6, n = 8) than the value of 9.3 ± 0.8 obtained in the previous experiment, as a consequence of a higher inoculum concentration. Nonetheless, this resulted in a N_gGsul_/N_gCpast_ ratio of 0.21 ± 0.06 (n = 8) very comparable to the value of 0.24 ± 0.04 obtained previously. This supports again that G*. sulfurreducens* was only able to grow during the *C. pasteurianum* growth phase. For these 8 experiments, the metabolite yields as function of *G.sulfurreducens* growth are displayed in Fig. 4. Significant correlations were found between the log-transformed *G. sulfurreducens* growth (log (Δcells.mL^−1^)) and the metabolite yields. A high *G. sulfurreducens* growth positively correlated with an increase of PDO (r = 0.95, p = 0.0002, n = 8), butyrate (r = 0.91, p = 0.0003, n = 8) and acetate (r = 0.93, p = 0.0069, n = 8). In contrast, butanol (r = −0.87, p = 0.0002, n = 8) and ethanol (r = −0.95, p = 0.0008, n = 8) were disfavoured when *G. sulfurreducens* cells increased. Overall, between 76 and 90% of total variance of the metabolite yields were explained by the growth of *G. sulfurreducens*.

## Discussion

*G. sulfurreducens* is a specialized microorganism with very limited metabolic capacities^27^,^28^. Amongst the metabolites found in fermentation and observed during this study, only acetate and H_2_ could have been used by *G. sulfurreducens* as potential electron donors, and acetate was the only available carbon source. The range of known electron acceptors that can be used by *G. sulfurreducens* is also limited, and consists in some metal ions, elemental sulfur, malate and fumarate^27^,^28^. None of these electron acceptors was present in the fermentation medium to sustain *G. sulfurreducens* growth (see Fig. 1). When growing together with *C. pasteurianum, G. sulfurreducens* could have also used glycerol fermentation end-products as sole electron acceptors. However, *G. sulfurreducens* was not able to grow after glycerol depletion, making the latter hypothesis invalid. The last alternative for *G. sulfurreducens* was to use *C. pasteurianum* as electron acceptor through mechanisms of interspecies electron transfers^1^,^2^,^4^,^29^,^30^,^31^. A similar behavior was previously reported in a co-culture of *Desulfovibrio vulgaris* and *Clostridium acetobutylicum* in which *D. vulgaris* was able to grow in presence of *C. acetobutylicum* and with no external electron acceptor^32^. Consistently, metabolic pattern of *C. acetobutylicum* was modified towards more electron dissipation via H_2_ evolution. Since *C. pasteurianum* DSM 525 was reported to be able to uptake extracellular electrons from a cathode during glycerol electro-fermentation^22^, it is highly probable that G*. sulfurreducens* transferred electrons to *C. pasteurianum*, either directly via interspecies wiring^1^ or indirectly using soluble electron mediators present in the medium such as L-cysteine^33^. Through such interaction, *G. sulfurreducens* triggered a significant metabolic shift for *C. pasteurianum* that enhanced the production of PDO at the expense of butanol and ethanol.

This metabolic shift could not be only due to direct dissipation of the electrons released by the growth of *G. sulfurreducens*. Instead, a non-linear relationship was found between *G. sulfurreducens* growth (*i.e.* electrons transferred to *C. pasteurianum*) and the change of production yields (Fig. 4). Extra electrons seemed to trigger metabolic regulations in favor of PDO pathway as main electron dissipation pathway. Once promoted, the PDO pathway could ensure intracellular redox balance by dissipating NADH, resulting in acetate and butyrate being produced from acetyl-CoA and butyryl-CoA instead of ethanol and butanol respectively. This would have theoretically led to more ATP production. Therefore a higher biomass production by *C. pasteurianum* could have been expected. In contrast, 39% less biomass production was observed in co-cultures than in pure cultures of *C. pasteurianum*. Consistently, Choi *et al*. (2014) reported a very similar drop of biomass production in glucose electro-fermentation: *C. pasteurianum* grown with a cathode as electron donor produced 41% less biomass than the open-circuit fermentation control^22^. The same phenomenon was also observed for other fermentative species growing in contact with a cathode^34^,^35^. If extracellular electrons uptake is an ATP-consuming process, then forcing electron consumption could be an interesting strategy to reduce biomass and by extension sludge formation during fermentation processes, leading to a better carbon and electron recovery. However, a better understanding of this decrease in biomass synthesis is necessary and should be the focus of further research.

In the present study, only 10 out of 16 co-culture experiments showed significant *G. sulfurreducens* growth, along with subsequent metabolic shift in glycerol fermentation. This observation could be related to the initial physiological state of *G. sulfurreducens* when inoculated. Indeed, a proteomic analysis previously revealed that under long-term terminal electron acceptor limiting conditions, *G. sulfurreducens* DSM 12127 enhanced the synthesis of some of its membrane-associated proteins^36^. In particular, this increased the synthesis of the pilA protein involved in nanowires conductivity^7^. The total heme content (*e.g.* cytochrome *c*) in the cells was also increased almost threefold compared with cells in midlog phase^36^. As a consequence, cells became poised and more susceptible to sense and use other electron acceptors that were encountered. In the present study, *G. sulfurreducens* pre-cultures were used as inoculum only after the cells precipitated as red aggregates (*i.e* with high heme content^36^). However the starvation time lasted only few days and the pre-cultures were probably heterogeneous and were constituted of both nanowire-rich aggregates and nanowire-poor planktonic cells. As the co-culture experiments duration was short, it is probable that only *G. sulfurreducens* cells that were already starved (*i.e* aggregates) at the inoculation time could have interacted with *C. pasteurianum*. That probably led to the heterogeneity in the observed results.

One option to ensure electrical connections during co-culture experiments could be to grow *G. sulfurreducens* as a biofilm on conductive materials. Studies focusing on electricity generation using *G. sulfurreducens* anodic biofilms have shown that such biofilms exhibit conductive properties through networks of nanowires^8^,^37^. Then, electron exchanges with a partner such as *C. pasteurianum* would be promoted as the two species would be physically connected through both pili networks and the conductive material^38^. This strategy has already been successfully applied to enhance DIET in methanogenesis^38^,^39^,^40^,^41^. For instance, inexpensive materials such as granular activated carbon were added into anaerobic digesters and resulted in an enrichment of *Geobacter*-like bacteria on the granules together with a significant increase of the methane production rate^41^. Moreover, attaching *G. sulfurreducens* on a carbon material could allow its use during continuous fermentation, thus overcoming the growth limitations observed during batch operations. In this case, the different growth rates of *G. sulfurreducens* and *C. pasteurianum* or other fermentative bacteria would not be an issue anymore and *G. sulfurreducens* wash-out would be avoided.

Under the hypothesis that interactions between *G. sulfurreducens* and *C. pasteurianum* are non-specific, continuous co-culture fermentations with electro-active and fermentative bacteria would provide many benefits such as: (i) recycling electrons from undesired fermentation end-products to promote the production more reduced compounds; (ii) avoiding the accumulation of inhibitors such as acetic acid; (iii) purifying the fermentation medium by removing undesired metabolites. Nonetheless, even if the present study is a very promising proof-of-concept, huge efforts on both process engineering and fundamental principles elucidation are required to fully take advantage of electro-active/fermentative interactions.

## Methods

### Bacterial strains

*G. sulfurreducens* DSM 12127 and *C. pasteurianum* DSM 525 were purchased from DSMZ (Braunschweig, Germany). Hungate techniques were used to cultivate the two microorganisms. 100 mL serum bottles sealed with butyl rubber stoppers and aluminum crimp caps were used with a working volume of 50 mL under a N_2_ gas phase (>99.995%). The DSMZ Medium 826 was used for *G. sulfurreducens* pre-cultures. The composition of the fermentation medium (per liter of water) used for *C. pasteurianum* pre-cultures was as follows: 10 g glycerol (≥99%), 0.50 g NH_4_Cl, 0.30 g KCl, 2.45 g NaH_2_PO_4_, 4.58 g Na_2_HPO_4_, 0.10 g Na_2_SO_4_, 0.15 g MgCl_2_.6H_2_O, 0.50 g L-cysteine, 10 mL vitamin solution (DSMZ medium 141) and 10 mL trace element solution (DSMZ medium 141). The medium was then adjusted at pH 6.5. All chemicals were purchased from Sigma-Aldrich (Saint-Louis, USA) at the highest grade available. Inoculated bottles were then stored and agitated in a room regulated at 35 °C.

### Fermentation medium and set-up

For all co-culture experiments and control experiments with *G. sulfurreducens* and *C. pasteurianum* cultivated alone, a minimal medium containing glycerol as a carbon and electron source for *C. pasteurianum* and acetate as an electron donor for *G. sulfurreducens* was used. Experiments were conducted using Hungate techniques and 100 mL serum bottles as described before. The composition of the fermentation medium (per liter of water) was as follows: 10 g glycerol (≥99%), 0.82 g Na-Acetate, 2.00 g NH_4_Cl, 0.75 g KCl, 2.45 g NaH_2_PO_4_, 4.58 g Na_2_HPO_4_, 0.28 g Na_2_SO_4_, 0.26 g MgCl_2_.6H_2_O, 2.90 mg CaCl_2_.2H_2_O, 0.50 g L-cysteine, 10 mL vitamin solution (DSMZ medium 141), 10 mL trace element solution (DSMZ medium 141) and 0.5 mL trace element solution SL-10 (DSMZ medium 320). The medium was then adjusted at pH 6.9. *C. pasteurianum* was inoculated by adding 1 mL of *C. pasteurianum* full grown pre-culture (dilution 1/50). *G. sulfurreducens* pre-cultures were used after the cells naturally precipitated into red aggregates (~7 days). Theses pre-cultures were centrifuged at 3600 g for 1 mn. Each pellet was then suspended in 2 mL of fresh co-culture medium (concentration x25). Finally, *G. sulfurreducens* was inoculated by adding 1 mL of this solution (final dilution 1/2). During experiments with different *G. sulfurreducens* inoculum concentrations, the concentrated inoculum solution was diluted using fresh co-culture medium prior to inoculation.

### Analytical methods

Concentrations of organic acids and alcohols were measured by HPLC with a refractive index detector (Waters R410). Samples were first centrifuged at 12,000 g for 15 min and then supernatants were filtered with 0.2 μm syringe filters. HPLC analysis were performed at a flow rate of 0.7 mL/min on an Aminex HPX-87H, 300 × 7.8 mm (Bio-Rad) column at a temperature of 35 °C. H_2_SO_4_ at 4 mM was used as the mobile phase.

Biogas composition was determined using a gas chromatograph (Clarus 580, Perkin Elmer) equipped with a thermal conductivity detector. The columns used were a RtQbond column (for H_2_, O_2_, N_2_ and CH_4_) and a RtMolsieve column (for CO_2_) and the carrier gas was argon at a pressure of 3.5 bar.

### Growth monitoring

Quantitative Real-Time polymerase chain reaction (qPCR) was used to follow *G. sulfurreducens* and *C. pasteurianum* growth. DNA was extracted with the Wizard^®^ Genomic DNA Purification Kit in accordance with the manufacturer’s instructions (Promega, Fitchburg, Wisconsin, USA). Extractions were verified using Infinite 200 PRO NanoQuant (Tecan Group Ltd., Männedorf, Zwitzerland). PCRs were prepared using 96-well real-time PCR plates (Eppendorf, Hamburg, Germany) and Biorad CFX96 (Biorad, Hercules, USA). Then, 12.5 μl of SsoAdvanced™ Universal SYBR^®^ Green Supermix, 5 μl of DNA extract with two appropriate dilutions, 250 nM forward primer (W406 5′-GGAAT AGCCT CCCGA AAGGG-3′ for *C. pasteurianum*, W410 5′-TGGGA AGTGC ATTGG AAACT G-3′ for *G. sulfurreducens*), 250 nM reverse primers (W407 5′-TCCAA CTAGC TAATG CGCCG-3′ for *C. pasteurianum*, W409 5′-GCGTC AGTAT CGGTC CAGAG-3′ for *G. sulfurreducens*), and water were added to obtain a final volume of 25 μl for all analyses.

An initial incubation of 2 min at 98 °C and 40 cycles of denaturation (95 °C, 15 s; 62 °C, 30 s) were performed. One standard curve was generated from each assay by using 10-fold dilutions in sterilized water (Gibco by Life Technologies) of a target plasmid (Eurofins Genomics, Germany). The average number of bacterial cells was calculated by dividing the average number of 16S rRNA gene copies per cell by a factor 9 for *C. pasteurianum*, and by a factor 2 for *G. sulfurreducens*^42^.

### Purity assessment of the co-cultures

Purity of the different cultures was checked using a capillary electrophoresis single-strand conformation polymorphism (CE-SSCP) analysis. The V3 regions of the 16S rRNA genes were amplified using the primers 330F (5′-AGGTCCAGACTCCTACGGG-3′) and 533R (5′-6FAM-TTACCGCGGCTGCTGGCAC-3′), which captures most of the bacterial diversity^43^. The PCR mixtures (25 μl) contained 0.25 μl of 2.5 U/μl Pfu Turbo DNA polymerase (Stratagene) with 2.5 μl of its corresponding buffer, 2 μl of 2.5 mM dNTP mixture, 0.4 mM of each primer, 10 ng of genomic DNA, and water were added to obtain a final volume of 25 μl. Reactions were performed in a Mastercycler thermal cycler (Eppendorf) as follows: 94 °C for 2 min, followed by 25 cycles of 94 °C for 30 sec, 61 °C for 30 sec, and 72 °C for 30 sec, with a final extension at 72 °C for 10 min. The amount and size of PCR products were determined using a Bioanalyzer 2100 (Agilent). Then samples were heat-denatured at 95 °C for 5 min and re-cooled directly in ice for 5 min. CE-SSCP electrophoresis was performed in an ABI Prism 3130 genetic analyzer (Applied Biosystems) in 50 cm capillary tubes filled with 10% glycerol, conformation analysis polymer and corresponding buffer (Applied Biosystems). Samples were eluted at 12 kV and 32 °C for 30 min, as described elsewhere^44^. CE-SSCP profiles were aligned with an internal standard (ROX) to consider the inter-sample electrophoretic variability. CE-SSCP profiles were normalized using the StatFingerprints library^45^ in R software version 2.9.2 (R. Development Core Team 2010).

### Statistical analysis

It was not possible to use parametric statistical tests such as t-test to compare the production yield means as the number of samples was too low. As a consequence, a two-sample Fisher-Pitman permutation test that does not require any distribution hypothesis was used. Two groups were compared (n = 4 and n = 2 resp.) with a total of 6 samples, meaning that 720 permutations could be generated. Therefore the p-values of the test were given at ±0.0014. The calculations were made using the “oneway_test” function of the package “coin” on R 3.1.3 software (R Development Core Team 2010).

Pearson correlations and significance calculations were also made with the R 3.1.3 software (R Development Core Team 2010). Prior to correlation calculations, the number of G*. sulfurreducens* cell produced during each co-cultures (Δcell/mL) were log transformed. For correlation coefficient calculations, the function “rcorr” of the package Hmisc was used. Significance levels were assessed using 9999 random permutations with the function “sample” of the package combinat (p-values ± 0.0001).

### N_g_ calculations

To evaluate the growth of a population, the number of time a population doubles (N_g_) can be used by calculating:


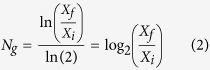


where X_i_ and X_f_ are the initial and final cell count respectively.

For each sample, 6 qPCR replicates were performed to assess the standard error of measurement of the technique. The raw qPCR results were log_2_ transformed before calculation of the variance between replicates. The standard error of measurement was calculated as:


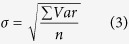


with Var being the variance between the replicates of one sample, and n the number of samples. A value of 0.23 log_2_(cells).mL^−1^ was found. As a consequence, it was considered that there were no growth in samples with N_g_ inferior to twice σ (~0.5 log_2_(cells).mL^−1^).

### Mass balance calculations

Carbon and electron mass balances were calculated by multiplying the molar amounts of the different chemicals by their carbon (C_eq_) and electron (E_eq_) molar equivalents as follows: Glycerol (C_eq_ = 3, E_eq_ = 14), PDO (C_eq_ = 3, E_eq_ = 16), Acetate (C_eq_ = 2, E_eq_ = 8), Ethanol (C_eq_ = 2, E_eq_ = 12), Butyrate (C_eq_ = 4, E_eq_ = 20), Butanol (C_eq_ = 4, E_eq_ = 24), H_2_ (C_eq_ = 0, E_eq_ = 2), Biomass (C_eq_ = 4, E_eq_ = 16).

The molar amount of biomass was calculated by multiplying the cell counts obtained from qPCR by the respective mass of dried cell. For each strain, the later was determined using 12 samples from a unique pre-culture. From the samples taken, 6 were used for the qPCR analyses (cell quantification) and the other 6 were freeze-dried (measurement of mass of dried cells). Final values of 2.25 10^−13^ g_dried mass_.cell^−1^ for *G. sulfurreducens*, and 1.09 10^−11^ g_dried mass_.cell^−1^ for *C. pasteurianum* were obtained. Finally, the total mass was converted in mole using a molecular formula of C_4_H_7_O_2_N for the dry mass^46^.

The acetate consumed by *G. sulfurreducens* was estimated using qPCR results and considering that 10% of the electron equivalents consumed by *G. sulfurreducens* was used for growth^9^. The remaining electron equivalents were considered to be transferred to *C. pasteurianum* as supplementary electron input (see Fig. 3).

Theoretical ATP generation was calculated by multiplying the molar amounts of the different fermentation end-products by their ATP yield as follows: PDO (Y_ATP/PDO_ = 0); Acetate (Y_ATP/Acetate_ = 2); Ethanol (Y_ATP/Ethanol_ = 1); Butyrate (Y_ATP/Butyrate_ = 3); Butanol (Y_ATP/Butanol_ = 2)^46^,^47^.

## Additional Information

**How to cite this article**: Moscoviz, R. *et al*. Cooperative growth of *Geobacter sulfurreducens* and *Clostridium pasteurianum* with subsequent metabolic shift in glycerol fermentation. *Sci. Rep.* **7**, 44334; doi: 10.1038/srep44334 (2017).

**Publisher's note:** Springer Nature remains neutral with regard to jurisdictional claims in published maps and institutional affiliations.

## Supplementary Material

Supplementary Information

## Figures and Tables

**Table 1:** Metabolite production yields and growth of the co-culture.

*G. sulfurreducens* biomass	*C. pasteurianum* biomass	Production yield (mmol/mol_Glycerol_ ± std)						n
Initial (cells/mL ± std)	N_gGsul_	N_gCpast_	PDO	Acetate	Butanol	Butyrate	Ethanol	
0	0	7.7 ± 0.3	159 ± 8	13 ± 2	233 ± 7	55 ± 3	106 ± 4	4
7.4 ± 2.8 10^5^	1.4 ± 0.3	7.1 ± 0.4	157 ± 10	12 ± 3	229 ± 8	56 ± 4	106 ± 8	4
8.5 ± 1.8 10^6^	1.0 ± 0.4	6.8 ± 0.3	170 ± 1	15 ± 3	220 ± 4	63 ± 2	85 ± 4	2*
1.2 ± 0.5 10^8^	1.7 ± 0.6	6.7 ± 0.4	194 ± 5	24 ± 2	209 ± 2	71 ± 5	63 ± 5	2*

^*^Only the results of experiments with a significant *G. sulfurreducens* growth are displayed (N_g_ > 0.5 log_2_(cells).mL^−1^).
