# Supplementary material for: Cooperative growth of Geobacter sulfurreducens and Clostridium pasteurianum with subsequent metabolic shift in glycerol fermentation
Source: Sci Rep. 2017 Mar 13;7:44334. doi: 10.1038/srep44334 (PMC5347079; doi:10.1038/srep44334)
Supplement: Supplementary Information [file srep44334-s1.pdf]

Supplementary information: Cooperative growth of *Geobacter sulfurreducens* and *Clostridium pasteurianum* with subsequent metabolic shift in glycerol fermentation

Roman Moscoviz, Florence de Fouchécour, Gaëlle Santa-Catalina, Nicolas Bernet and Eric Trably*

*Corresponding author: *eric.trably@inra.fr*

LBE, INRA, 102 Avenue des étangs, 11100 Narbonne, France

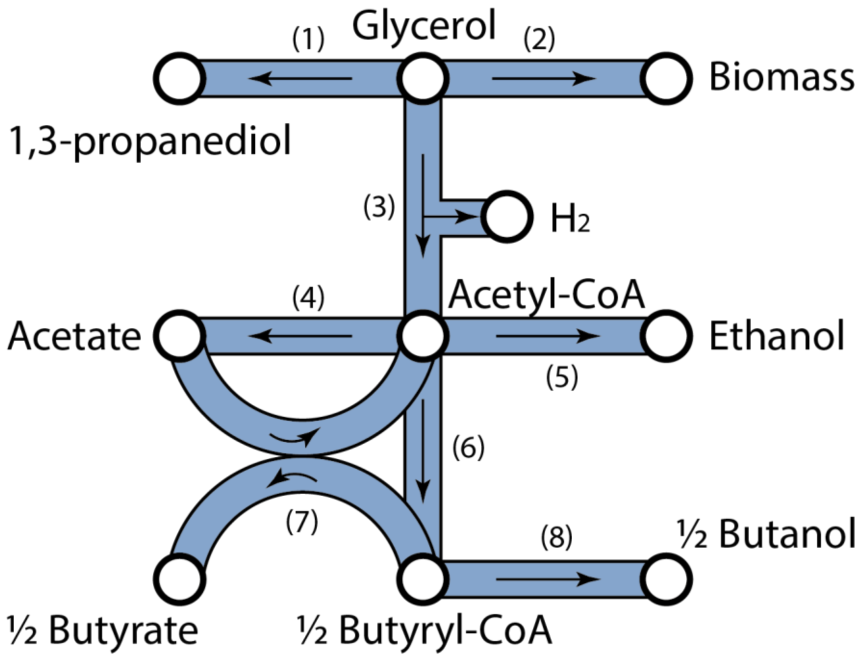

Figure S1: Simplified catabolic pathways of glycerol fermentation by *Clostridium pasteurianum*.

Table S1: Condensed metabolic pathways of glycerol fermentation

**Reaction**	**Legend**	**Ref**
Glycerol + NADH → PDO + H_2O	(1)	1,2
Glycerol + ¾ NH_3 + 7.5 ATP + 6 H_2O → ¾ $C_4H_7O_2N$ + NADH	(2)	1
Glycerol + CoA → Acetyl-CoA + CO_2 + H_2 + ATP + H_2O + 2 NADH	(3)	1,2
Acetyl-CoA → Acetate + ATP + CoA + H_2O	(4)	1–3
Acetyl-CoA + 2 NADH → Ethanol + CoA	(5)	1–3
2 Acetyl-CoA + 2 NADH → Butyryl-CoA + CoA + H_2O	(6)	3–5
Butyryl-CoA + Acetate → Butyrate + Acetyl-CoA	(7)	3–5
Butyryl-CoA + 2 NADH → Butanol	(8)	6,7

For more readability, NAD^+, H^+ and ADP are omitted in the presented equations. $C_4H_7O_2N$ correspond to the mean raw formula of bacterial biomass[1].

Table S2: Redox and ATP balanced reactions of glycerol metabolism

**Global reaction**	**ΔrG°' ($kJ.mol_{glycerol}^{-1}$)**
53 Glycerol + 3 NH_3 → 3 $C_4H_7O_2N$ + 15 Acetate + 15 CO_2 + 15 H_2 + 34 PDO + 25 H_2O	-52.2
38 Glycerol + 3 NH_3 → 3 $C_4H_7O_2N$ + 30 Ethanol + 30 CO_2 + 30 H_2 + 4 PDO + 10 H_2O	-50.1
48 Glycerol + 3 NH_3 → 3 $C_4H_7O_2N$ + 10 Butyrate + 20 CO_2 + 20 H_2 + 24 PDO + 40 H_2O	-109.1
38 Glycerol + 3 NH_3 → 3 $C_4H_7O_2N$ + 15 Butanol + 30 CO_2 + 30 H_2 + 4 PDO + 25 H_2O	-92.3

Equations were balanced using equations from Table S1. Standard Gibbs free energy of reaction (ΔrG°', for pH 7 and T = 25 °C) were calculated using Gibbs free energy of formation from Kleerebezem and Van Loosdrecht (2010)[8].

Bibliography

1. Zeng, A.-P., Biebl, H., Schlieker, H. & Deckwer, W.-D. Pathway analysis of glycerol fermentation by Klebsiella pneumoniae: regulation of reducing equivalent balance and product formation. *Enzyme Microb. Technol.* **15,** 770–779 (1993).
2. Zeng, A.-P. Pathway and kinetic analysis of 1, 3-propanediol production from glycerol fermentation by Clostridium butyricum. *Bioprocess Eng.* **14,** 169–175 (1996).
3. Temudo, M. F., Kleerebezem, R. & van Loosdrecht, M. Influence of the pH on (open) mixed culture fermentation of glucose: A chemostat study. *Biotechnol. Bioeng.* **98,** 69–79 (2007).
4. Louis, P. & Flint, H. J. Diversity, metabolism and microbial ecology of butyrate-producing bacteria from the human large intestine. *FEMS Microbiol. Lett.* **294,** 1–8 (2009).
5. Vital, M., Howe, A. C. & Tiedje, J. M. Revealing the bacterial butyrate synthesis pathways by analyzing (meta) genomic data. *MBio* **5,** e00889–14 (2014).
6. Atsumi, S. *et al.* Metabolic engineering of Escherichia coli for 1-butanol production. *Metab. Eng.* **10,** 305–311 (2008).
7. Jin, C., Yao, M., Liu, H., Lee, C. F. & Ji, J. Progress in the production and application of n-butanol as a biofuel. *Renew. Sustain. Energy Rev.* **15,** 4080–4106 (2011).
8. Kleerebezem, R. & Van Loosdrecht, M. C. M. A Generalized Method for Thermodynamic State Analysis of Environmental Systems. *Crit. Rev. Environ. Sci. Technol.* **40,** 1–54 (2010).
